# Associations of aortic stiffness and intra-aortic flow parameters with epicardial adipose tissue in patients with type-2 diabetes

**DOI:** 10.3389/fcdhc.2023.1106342

**Published:** 2023-05-26

**Authors:** Khaoula Bouazizi, Mohamed Zarai, Abdallah Noufaily, Mikaël Prigent, Thomas Dietenbeck, Emilie Bollache, Toan Nguyen, Valéria Della Valle, Eléonore Blondiaux, Karine Clément, Judith Aron-Wisnewsky, Fabrizio Andreelli, Alban Redheuil, Nadjia Kachenoura

**Affiliations:** ^1^ Laboratoire d’Imagerie Biomédicale (LIB), Sorbonne Université, Institut National de la Recherche Médicale (INSERM), Centre National de la Recherche Scientifique (CNRS), Paris, France; ^2^ ICAN Imaging, Institute of Cardiometabolism and Nutrition (ICAN), Paris, France; ^3^ Unité d’Imagerie Cardiovasculaire et Thoracique (ICT), Pitié-Salpêtrière Hospital, Paris, France; ^4^ Assistance Publique Hôpitaux de Paris, Radiology Department, Armand-Trousseau Hospital, Paris, France; ^5^ Sorbonne Université, INSERM, Nutrition and Obesities; approches systémiques (NutriOmique), Pitié-Salpêtrière Hospital, Nutrition Department, Paris, France; ^6^ Assistance Publique Hôpitaux de Paris, Nutrition Department, Centre de Recherche en Nutrition Humaine (CRNH) Ile-de-France, Pitié-Salpêtrière Hospital, Paris, France; ^7^ Assistance Publique Hôpitaux de Paris, Diabetology Department, Pitié-Salpêtrière Hospital, Paris, France

**Keywords:** epicardiac adipose tissue, cardiovascular MRI, type 2 diabetes, aortic flow, cardiometabolic disease

## Abstract

**Background:**

It has been shown that increased aortic stiffness is related to type-2 diabetes (T2D) which is considered as a risk factor for cardiovascular disease. Among other risk factors is epicardial adipose tissue (EAT) which is increased in T2D and is a relevant biomarker of metabolic severity and adverse outcome.

**Purpose:**

To assess aortic flow parameters in T2D patients as compared to healthy individuals and to evaluate their associations with EAT accumulation as an index of cardiometabolic severity in T2D patients.

**Materials and methods:**

Thirty-six T2D patients as well as 29 healthy controls matched by age and sex were included in this study. Participants had cardiac and aortic MRI exams at 1.5 T. Imaging sequences included cine SSFP for left ventricle (LV) function and EAT assessment and aortic cine and phase-contrast imaging for strain and flow parameters quantification.

**Results:**

In this study, we found LV phenotype to be characterized by concentric remodeling with decreased stroke volume index despite global LV mass within a normal range. EAT was increased in T2D patients compared to controls (p<0.0001). Moreover, EAT, a biomarker of metabolic severity, was negatively correlated to ascending aortic (AA) distensibility (p=0.048) and positively to the normalized backward flow volume (p=0.001). These relationships remained significant after further adjustment for age, sex and central mean blood pressure. In a multivariate model, presence/absence of T2D and AA normalized backward flow (BF) to forward flow (FF) volumes ratio are both significant and independent correlates of EAT.

**Conclusion:**

In our study, aortic stiffness as depicted by an increased backward flow volume and decreased distensibility seems to be related to EAT volume in T2D patients. This observation should be confirmed in the future on a larger population while considering additional biomarkers specific to inflammation and using a longitudinal prospective study design.

## Introduction

Type-2 diabetes mellitus (T2D) is a prevalent disease associated with increased cardiovascular risk factors. Part of this increased hazard is related to peripheral and central arteries stiffness and disease, as described in the study of Sharif et al. conducted on a sub-population of the Second Manifestations of ARTerial disease (SMART) cohort on T2D patients (1). Arterial stiffness, defined as a loss in arterial wall elasticity, is associated with high blood pressure (2) and flow disturbance (3). Indeed, increased arterial stiffness is specifically associated with increased systolic and pulse pressure (augmented pressure) as well as increased wave reflections, which ultimately contribute to adverse left ventricular remodeling (4). Large artery stiffness in diabetic patients is often explained by intricated processes including wall thickening by advanced glycation end-product formation with collagen cross-linking (5), endothelial dysfunction with nitric oxide dysregulation (6) and vascular calcification, which have been described in both type-1 diabetes (7) and T2D (8). While arterial stiffness has been widely described in T2D patients using conventional biomarkers such as pulse wave velocity (PWV) and distensibility (9–11), there are still limited data on potential intra-aortic flow alterations in these patients. Changes in aortic flow patterns could be related to increased aortic wall stress and ventricular afterload and decreased coronary flow potentially aggravating left ventricle (LV) remodeling and its susceptibility to ischemia. In particular, the backward flow (BF) volume, which was shown to significantly increase in magnitude and occur earlier within the cardiac cycle with increasing age and to be associated with changes in aortic geometry and pressure wave reflections (3) in the general population have not been widely studied in T2D patients. Indeed, BF parameters could be considered as early functional markers of subclinical impairment of circulatory efficiency in diabetes.

Excessive or abnormal accumulation of epicardial adipose tissue (EAT) is associated with metabolic diseases and cardiovascular complications (12, 13). Recent studies suggest that EAT thickness is associated with worse cardiac performance and multiple traits of subclinical cardiac systolic dysfunction in T2D patients (14). Indeed, EAT is an active endocrine tissue which can significantly influence some chronic conditions (15). EAT can act both locally or by paracrine secretion of mediators including adipokines, inflammatory cytokines, or reactive oxidative species that can potentially adversely affect the adjacent coronary vessels and myocardium. Since EAT is metabolically active, secreting numerous substances associated with cardiovascular injuries such as TNFα, IL-6 and IL-1β (16), it may imply endocrine systemic effects of EAT (17), but could also have a systemic effect on aortic hemodynamics through EAT-mediated metabolic and inflammatory pathways. Exploring the link between EAT and aortic hemodynamics might provide a better understanding on how EAT may be considered in the future as a marker of cardiovascular risk in T2D.

While computed tomography (CT) has proven useful in quantifying EAT (18), it induces patient radiation exposure. To date, most studies have been using CT (19, 20). Recently, MRI has emerged as a non-invasive and effective imaging modality for identifying whole body fat including ectopic fat depots (21). Specifically, cine Steady-State Free Precession (SSFP) MRI provides a high signal-to-noise ratio and optimal blood/myocardial/fat contrasts to enable a precise delineation of the endocardial, epicardial and pericardial borders. Another advantage of MRI resides in its ability to capture within the same exam, heart and arterial wall dynamics as well as phase-contrast sequence-derived circulating blood flow velocities, which could help study complex pathophysiological interactions and disentangle associations of surrounding adipose tissue with wall dynamics (22) and inner flow disturbance (3).

Accordingly, our aims were to assess changes in aortic stiffness and flow parameters in T2D patients as compared to healthy individuals and to evaluate their associations with EAT accumulation, as an index of cardiometabolic severity (23, 24).

## Materials and methods

### Study population

We studied 65 individuals (31 females, age: 53 ± 11 years), a subgroup from the Metagenomics in Cardiometabolic Diseases (FP7 Metacardis) study, including 29 healthy subjects and 36 T2D patients who underwent an MRI exam. Healthy subjects were matched according to age ( ± 5 years) and sex with T2D patients. All subjects provided informed consent and the study protocol was approved by the local Institutional Review Board. Further details including inclusion criteria are provided online (https://clinicaltrials.gov/ct2/show/NCT02059538).

### Laboratory parameters

Laboratory parameters were assessed using standard analytical methods. Hypertension was diagnosed by a systolic/diastolic blood pressure >140/90 mmHg and or a prescribed anti-hypertensive treatment and T2D was diagnosed by a fasting blood glucose > 7.0 mmol/l, a random glucose measurement > 11.0 mmol/l or a HbA1c > 6.5% or a prescribed glucose-lowering drug, both defined according to the American Diabetes Association criteria.

Blood samples were collected after an overnight fast. Fasting serum glucose, triglycerides, and HbA1c were measured using enzymatic methods. Fasting serum insulin and C-peptide were measured using a chemiluminescence assay (Insulin Architect, Abbott). High-sensitivity C-reactive protein (CRP) was measured using an IMMAGE automatic immunoassay system (Beckman-Coulter) and high-sensitivity interleukin 6 (hs-IL-6) was measured using the Human IL-6 Quantikine HS ELISA Kit (R&D Systems). IFN-γ–induced protein 10 (IP-10), interleukin 7 (IL-7), macrophage migration inhibitory factor (MIF), C-X-C motif chemokine ligand 2 (CXCL2), and 5 (CXCL5) were measured by using a Luminex assay (ProcartaPlex Mix&-Match Human 13-plex; eBioscience, San Diego, CA, USA).

### MRI acquisitions

MRI data were obtained using a 1.5 T scanner (MAGNETOM Aera, Siemens Healthineers, Erlangen, Germany) using a thoracic phased-array surface coil with ECG gating. A cine SSFP sequence was used for short- and long-axis cardiac imaging with the following typical scan parameters: echo time (TE)=1.18 ms, temporal resolution=52 ms, acquisition matrix=288x288, slice thickness=8 mm, pixel spacing=1.56 mm², flip angle=58° and typical Field-of-View (FOV)=44.9 cm². The short axis stack covered the left ventricle from base to apex.

Aortic imaging included a cine SSFP sequence acquired transverse to the ascending and descending thoracic aorta at the level of the pulmonary artery bifurcation, with the following imaging parameters: TE=1.7 ms, temporal resolution=26.9 ms, acquisition matrix=290x352, slice thickness=8 mm, pixel spacing=1.08 mm², flip angle=74° and typical FOV=31.3 x38 cm². A 2D gradient echo phase-contrast (PC) sequence with through-plane velocity-encoding was applied at the same location as for the aortic cine SSFP sequence. Imaging parameters were: TE=2.3 ms, temporal resolution=35 ms, maximal velocity encoding=150 cm/sec; slice thickness=8 mm, pixel spacing=1.77 mm², flip angle=60°, temporal resolution=18 ms.

Blood pressures were measured at the beginning and the end of the MRI exam as well as during the 2D PC aortic imaging while the patient was lying in supine position within the magnet, using an MRI compatible device (SphygmoCor^®^; AtCor Medical, West Ryde, NSW, Australia). Blood pressure waveform was then averaged from these three measurements to collect systolic/diastolic pressures and subsequently pulse pressure (PP). Brachial pressures were also measured during the inclusion visit.

### Cardiac function evaluation and adipose tissue segmentation

**Figure 1:**
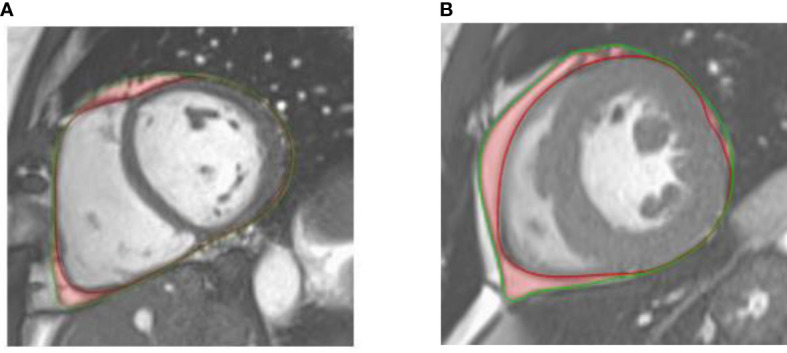
Illustration of epicardial adipose tissue (outlined in red shading) on an end-diastolic mid-ventricular short axis cine-SSFP image of a healthy control **(A)** and a patient with type 2 diabetes **(B)**. The epicardium and pericardium are highlighted with red and green lines, respectively.

Left ventricular (LV) end-diastolic mass (LVM) as well as LV and epicardial adipose tissue volumes were estimated from cine SSFP data using a commercial software (QMass, Medis, Leiden, The Netherlands, version 4.0.24.4). LV end-diastolic (EDV) and end-systolic (ESV) volumes were obtained by manually tracing the LV endocardial and epicardial borders on the stack of short-axis images covering the whole heart. Ejection fraction (EF), stroke volume (SV) and cardiac output (CO) were further calculated. LV EDV, ESV, SV, CO and mass were indexed to body surface area (BSA). EAT was defined as the adipose tissue between the epicardium and the pericardium. EAT area was measured with manual contouring from consecutive short-axis images in end-diastole (**Figure 1**), while including only slices with a full circular section of the LV myocardium and avoiding apical segment to guarantee measurement homogeneity across subjects and avoid artifacts or partial volume effects in extreme slices. The resulting EAT volume was then converted into mass (considering the average fat density of 0.9 g/cm^3^) (25) and indexed to BSA.

### Aortic geometry, stiffness and flow indices

The ARTFUN software (LIB, Sorbonne Université) (26) was used to automatically measure: 1) the ascending aorta (AA) and descending aorta (DA) cross-sectional areas from cine SSFP images of the aorta and 2) AA and DA global, forward (FF) and backward (BF) flow volumes and peaks from 2D PC MRI images of the aorta, as described in (3). Aortic distensibility was calculated as:


$$Distensibility\;\left(mmHg^{-1}\right)\;=\;\frac{Strain}{PP}$$

where *Strain* is the difference between the maximal and minimal cross-sectional aortic area over the cardiac cycle divided by the minimal cross-sectional aortic area, *PP* being the central pulse pressure (in mmHg).

### Statistical analysis

Statistical analyses were performed using GraphPad PRISM 6 (GraphPad Software Inc., Canada). Continuous variables are expressed as means ± standard deviations Student's t-test. For comparisons between groups, a Student’s t-test was performed. Correlation coefficients for linear regression between continuous variables were provided. Multivariate regression models were used to evaluate the relationships between indexed EAT and aortic parameters adjusted for covariates with known confounding effects on aortic function, such as age, sex, and mean blood pressure (27). EAT inter-observer reproducibility and intra-observer reproducibility after 4 weeks was evaluated on a subgroup of 20 and 10 random individuals including controls and T2D patients, respectively. Bland-Altman plots were used to compare independent measures, while providing mean absolute differences and limits of agreement. Coefficients of variation (CV) were further calculated between the repeated measurements of EAT, as the standard deviation of the differences between measurements divided by their mean. The significance was set to *p*<0.05 for all tests.

## Results

### Basic characteristics

Patient clinical characteristics as well as LV parameters are detailed in **Table 1** for both T2D patients and healthy controls. As expected, the biochemical parameters related to diabetes were markedly higher in T2D patients compared to controls (**Table 1**). BMI, BSA, heart rate (HR) and prevalence of hypertension were significantly higher in the T2D group than in controls (p<0.001).

**Table 1:** Clinical characteristics of the study population.

	Controls	T2D	*p*-value
n	29	36	
Male/Female	18/11	16/20	0.16
Age (years)	51.52 ± 10.96	54.66 ± 9.90	0.23
BMI (kg/m^2^)	23.05 ± 1.26	32.00 ± 5.71	<0.0001
BSA (m^2^)	1.77 ± 0.14	1.99 ± 0.21	<0.0001
Hypertension, number (%)	0 (0%)	15 (42%)	0.0001
Brachial SBP (mmHg)	128.1 ± 17.53	135.8 ± 16.30	0.08
Brachial DBP (mmHg)	77.96 ± 10.32	82.61 ± 13.20	0.14
HR (bpm)	61.64 ± 9.11	71.64 ± 13.60	0.001
Smoking, number (%)	1 (3%)	1 (2%)	0.89
Biochemical parameters			
Adiponectin (mg/l)	5.42 ± 2.45	3.97 ± 2.02	0.03
Leptin (ng/ml)	8.31 ± 9.82	25.18 ± 23.84	0.01
Plasma creatinin (µmol/l)	80.9 ± 12.87	74.78 ± 14.38	0.12
CRP (mg/l)	2.29 ± 2.15	3.46 ± 6.16	0.47
CXCL5 (pg/ml)	537 ± 632	1688 ± 3422	0.2
Eotaxin (pg/ml)	21 ± 15	23 ± 19	0.66
IP-10 (pg/ml)	23 ± 13	27 ± 27	0.59
HDL-cholesterol (mmol/l)	2 ± 1.04	1.32 ± 0.8	0.009
LDL-cholesterol (mmol/l)	2.75 ± 0.84	2.8 ± 0.99	0.8
Triglycerides (mmol/l)	0.83 ± 0.59	1.61 ± 0.9	0.001
Glycated hemoglobin (HbA1c) (%)	5.54 ± 0.31	7.71 ± 1.5	<0.0001
Fasting insulin (mmol/l)	5.64 ± 3.68	14.51 ± 10.63	0.0031
Antihypertensive intake (%)	0	39%	
Insulin intake	0	16%	
Metformin intake	0	75%	
LV parameters			
LVM (g)	95.16 ± 26.20	113.8 ± 36.88	0.02
LVM/BSA (g/m^2^)	53.46 ± 11.64	56.66 ± 15.37	0.37
LV ESV/BSA (ml/m^2^)	35.33 ± 10.61	29.75 ± 11.20	0.05
LV EDV/BSA (ml/m^2^)	87.58 ± 16.32	71.38 ± 16.05	0.0003
LVM/LV EDV (g/ml)	0.62 ± 0.14	0.79 ± 0.19	0.0005
LVEF (%)	61.13 ± 7.28	59.93 ± 9.82	0.59
SV/BSA (ml/m^2^)	53.89 ± 11.16	41.50 ± 9.76	<0.0001
CO/BSA, or cardiac index (l/min/m^2^)	3.32 ± 0.68	2.99 ± 0.60	0.04
Epicardial adipose tissue			
EAT (g/m^2^)	9.93 ± 3.34	23.71 ± 9.03	<0.0001

BMI, body mass index; BSA, body surface area; SBP/DBP, systolic/diastolic blood pressure; HR, heart rate; bpm, beats per minute; LVM, left ventricular mass; LV ESV, left ventricular end-systolic volume; LV EDV, left ventricular end-diastolic volume; SV, stroke volume; LVEF, left ventricular ejection fraction; CO, cardiac output; EAT, epicardial adipose tissue. The cardiac index is the cardiac output indexed to the BSA. CRP, C-reactive protein; HDL-C, High-density lipoprotein cholesterol; LDL-C, Low-density lipoprotein cholesterol.

There was no effect of treatment (insulin and/or metformin) on EAT volume in diabetic patients.

There was a trend towards higher LV mass in T2D patients as compared to controls, but such increase did not reach statistical significance after indexation to BSA. Indexed end-diastolic volume and stroke volume were significantly lower in T2D patients, as compared to controls. Concentric LV remodeling was significantly more pronounced in T2D patients than in controls, as revealed by the higher LV mass to end-diastolic volume ratio.

**Figure 2:**
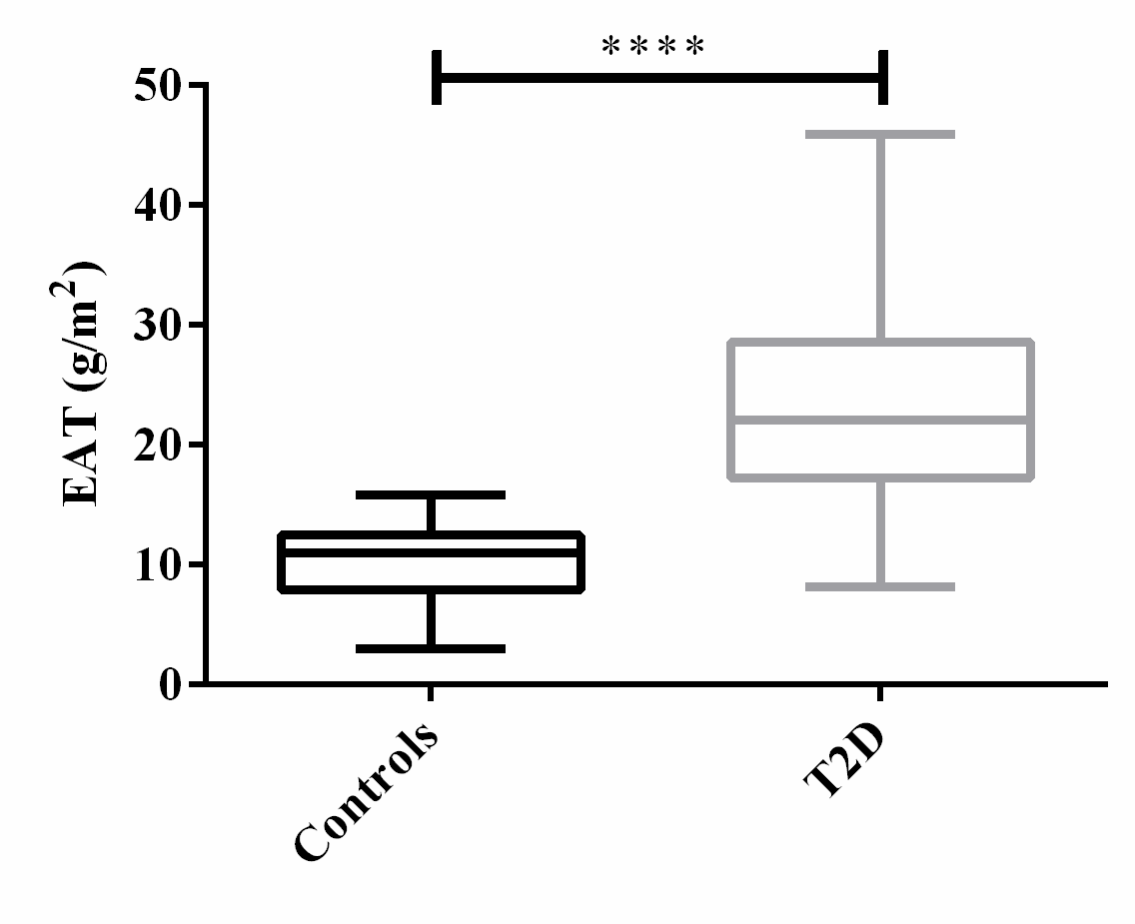
Distribution of EAT among T2D patients and controls. ****: p<0.0001.

As expected, indexed EAT was significantly higher in T2D patients than in controls (**Figure 2**), by more than 2 folds on average, even after adjustment for age, sex and BMI. Of note, EAT measurement was reproducible with an intra-operator CV of 4% and an inter-operator CV of 6%. Bland-Altman intra- and inter-observer mean bias [limits of agreement] were, respectively, equal to -0.1 [-0.76– 0.56] g/m^2^ and 0.23 [-1.8 – 2.2] g/m^2^.

While EAT was not associated with age in the entire study group (p=0.10), EAT was positively and significantly correlated (r = 0.52, p<0.0001) to BMI. Increased EAT volume was related to poor glycemic control over time as measured by glycated hemoglobin (HbA1C) with an overall EAT expansion of 6.3 g/m^2^ by 1% increase of HbA1C.

### Aortic geometry, flow and stiffness measurements

**Figure 3:**
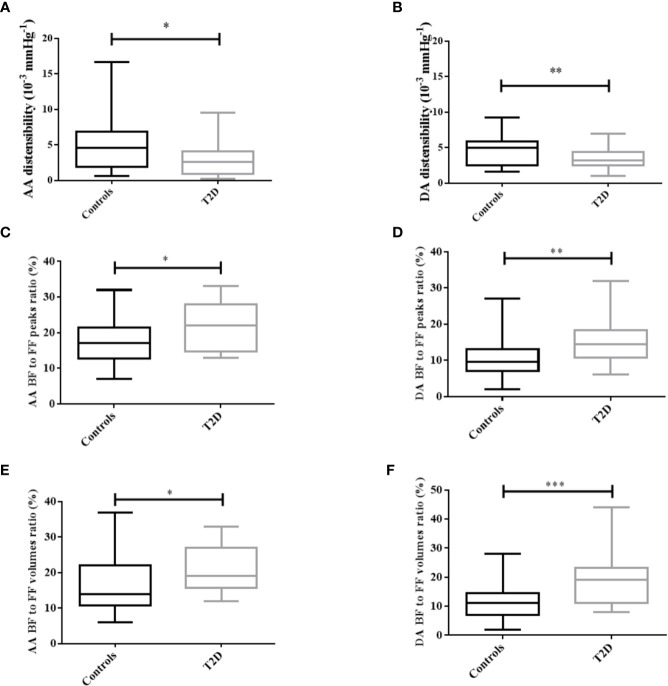
Distribution of aortic distensibility **(A, B)** and flow parameters **(C–F)** in T2D patients and controls, for both the ascending (AA) and descending aorta (DA). FF/BF: forward/backward flow. *: p<0.05, **: p<0.01, ***: p<0.001.

Aortic parameters are detailed in **Table 2** for both T2D patients and controls. T2D patients have slightly higher central blood pressures than controls, although such changes did not reach statistical significance (p≥0.05). Both AA and DA were slightly dilated and significantly stiffer in T2D than in controls, as revealed by higher aortic areas as well as a lower distensibility (p<0.01, **Figures 3A, B**). While there were no significant changes in FF volumes and peaks in both AA and DA in T2D patients than in controls, there is a significant increase in the proportion of BF at both locations, as revealed by higher BF to FF ratios in terms of peaks (**Figures 3C, D**) and volumes (**Figures 3E, F**).

**Table 2:** Aortic parameters of the studied population.

	Controls	T2D	*p*-value
Central SBP (mmHg)	114 ± 17	122 ± 15	0.05
Central DBP (mmHg)	78 ± 11	84 ± 14	0.08
Central PP (mmHg)	37 ± 1	38 ± 1	0.50
Aortic geometry and stiffness indices			
AA min area (cm^2^)	6.95 ± 1.6	8.05 ± 2	0.02
DA min area (cm^2^)	3.8 ± 0.9	4.5 ± 1	0.01
AA strain (%)	17 ± 12	11 ± 9	0.02
DA strain (%)	16 ± 5	13 ± 6	0.02
AA distensibility (10^-3^ mmHg^-1^)	5.3 ± 3.8	3.1 ± 2.5	0.01
DA distensibility (10^-3^ mmHg^-1^)	5 ± 2.3	3.3 ± 1.5	0.001
Aortic flow indices			
AA GF total volume (ml)	84 ± 20	76 ± 19	0.10
AA BF to FF peak ratio (%)	17 ± 6	22 ± 7	0.02
AA BF to FF systolic volume ratio (%)	7.26 ± 5	9.36 ± 6.5	0.9
AA BF to FF total volume ratio (%)	16 ± 8	21 ± 6	0.01
DA GF total volume (ml)	52 ± 12	45 ± 12	0.03
DA BF to FF peak ratio (%)	10 ± 5	15 ± 6	0.002
DA BF to FF systolic volume ratio (%)	3.19 ± 2	5.2 ± 3.6	0.014
DA BF to FF total volume ratio (%)	12 ± 6	19 ± 9	0.0009

SBP/DBP, systolic/diastolic blood pressure during MRI; PP, pulse pressure; AA/DA, ascending/descending aorta; GF/FF/BF, global/forward/backward flow; V_BF_/V_FF_, backward/forward flow volume.

### Associations of EAT with aortic stiffness and flow indices

**Figure 4:**
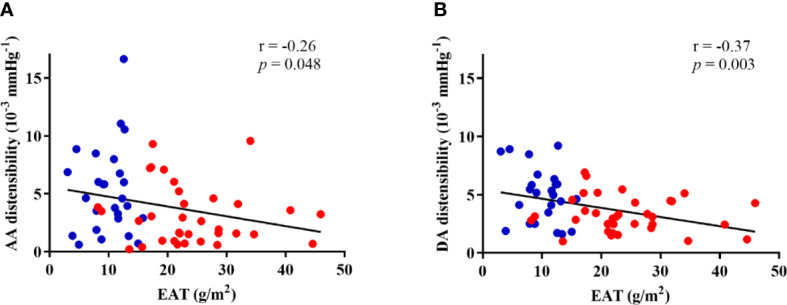
Correlations of EAT with ascending (AA) **(A)** and descending (DA) **(B)** aortic distensibility in both groups. Controls are presented in blue dots and T2D patients are presented in red dots.

Negative and significant correlations were observed between EAT and both AA (r=-0.26, p=0.048) and DA (r=-0.37, p=0.003) distensibility (**Figure 4**) in the overall population.

**Figure 5:**
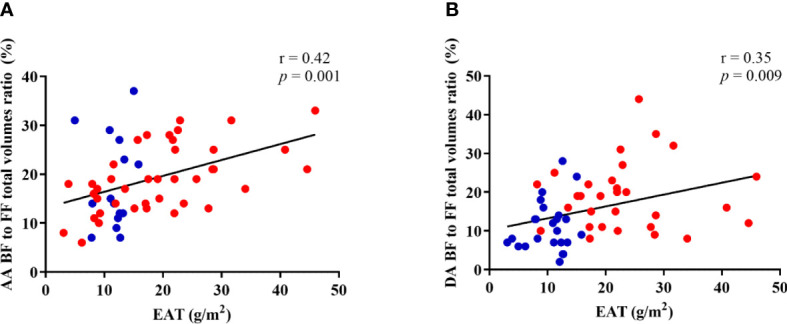
Correlation between EAT and ascending **(A)** and descending **(B)** aorta backward (BF) to forward (FF) flow volumes ratio. Controls are presented in blue dots and T2D patients are presented in red dots.

EAT was positively and significantly correlated to the BF to FF volumes ratio (**Figure 5**) in the entire study group for both the AA (r=0.42, p=0.001) and DA (r=0.35, p=0.009). Of note, ascending aortic BF to FF volumes ratio was also positively and significantly correlated to EAT in T2D patients alone (r=0.38, p=0.03). Such relationships remained significant after further adjustment for age, sex and central mean blood pressure. In a multivariate model presence/absence of T2D and AA backward (BF) to forward (FF) flow volumes ratio are both significant and independent correlates of EAT.

### Associations between aortic parameters and biochemical biomarkers and treatments

Significant relationships were observed between aortic flow and stiffness parameters and inflammatory biomarkers. Interestingly, a positive relationship between the AA systolic BF to FF volumes ratio and CXCL5 (r=0.36, p=0.01) was found. Ascending aortic BF to FF volumes ratio was also significantly associated to eotaxin (r=0.32, p=0.045) and to MIF (r=-0.37, p=0.01). Regarding AA stiffness indices, we found a significant correlation between IP-10 and aortic distensibility (r=0.96, p<0.0001) and between CRP and aortic strain (r=0.35, p=0.017).

Of note, there was no significant difference in aortic stiffness and flow parameters according to antihypertensive or antidiabetic treatment subgroups.

## Discussion

Our main finding was a positive relationship between aortic functional alterations, reflected by increased aortic stiffness and flow disturbances, and higher metabolic risk profile defined by increased EAT volume in T2D patients. We specifically found that T2D patients had a significantly stiffer thoracic aorta than controls, in agreement with previous literature (28, 29). Indeed, aortic strain and distensibility were both lower in T2D patients compared to healthy controls (30, 31), also in agreement with a recent report albeit using echocardiography of the aortic root instead of tubular ascending aorta (32). In the present study, we propose a comprehensive functional and hemodynamic evaluation of the proximal aorta as provided by MRI. We show that local aortic distensibility and local flow parameters such as AA and DA backward to forward flow peak and volume ratio were also altered in T2D patients.

In this study, EAT was segmented using cine SSFP MRI images which are typically acquired in standard cardiac MRI exams. Cine SSFP images can distinguish the adipose tissue from the blood pool and myocardium allowing for EAT delineation. Amount of EAT, known to be increased in T2D (33), is valuable for metabolic studies since it serves as a good surrogate of metabolic and associated cardiovascular risks. It may serve as an effective tool for the diagnosis and stratification of cardiovascular risk profiles in patients with metabolic diseases and has been associated to incident myocardial or cerebral infarction and heart failure (34).

In our study, T2D patients had increased EAT volume compared to controls, which remained significant after adjusting for the confounding effects of age, sex and BMI, revealing an independent association between EAT and T2D. It is separately known that EAT is increased in T2D and is a relevant biomarker of metabolic severity and adverse outcome (23).

We found LV phenotype in T2D patients to be characterized by concentric remodeling with decreased SV index despite global indexed LV mass within a normal range. We observed that EAT was significantly associated with BMI, which is in line with previous findings (35, 36).

Our data demonstrate a significant and negative correlation between EAT and aortic strain and distensibility and a positive correlation between EAT and normalized backward flow. These findings suggest that the amount of EAT, which is a biomarker of metabolic severity, is related to the degree of local aortic stiffness and aortic flow alterations (37). To the best of our knowledge, this relationship was previously established in healthy volunteers but not in T2D patients (38).

EAT is not an inert structure but is a highly active endocrine organ with anabolic and deleterious secretome through the action of adipokines, activated fibrocytes and cytokines (39). This endocrine activity may result in both systemic and local inflammatory responses, and endothelial dysfunction shared with the physiopathology of aortic backward flow. Indeed, Doğan et al. (22) reported a relationship between EAT and aortic stiffness using echocardiography in patients with primary hypertension. The elastic properties of the arterial wall as defined by two major proteins (elastin and collagen) are altered with aging and accelerated in the presence of risk factors such as diabetes over a lifespan, resulting in increased arterial stiffness. Hemodynamic associates of arterial stiffness include increased central systolic pulse pressure, increased backward flow volume (3) and impaired myocardial perfusion.

Our hypothesis was that increased EAT amount may indirectly facilitate aortic degeneration through chronic inflammation pathways. In this study we did not find significant relationships between EAT and circulating biomarkers of inflammation potentially owing to the small sample size and lack of statistical power. Our study adds to the body of evidence on the potential associations between EAT and arterial alteration in T2D. Most reported studies have focused on carotid arteries (40) and to a lesser extent on the aorta. An important property of EAT, in addition of being a visceral fat tissue making it more deleterious, resides in the fact that, because of its location, it can interact locally with coronary arteries and the myocardium through paracrine and/or vasocrine pathways (16). Park et al. (41) showed that EAT correlated with arterial stiffness as measured by cardio-ankle vascular index. Although the relationship between EAT and increased arterial stiffness was demonstrated in previous works, studies investigating the effects of EAT on aortic flow disturbance in patients with T2D using MRI have not been performed yet. In T2D when LVEF is still preserved and without overt LV hypertrophy, increased aortic stiffness and related flow reversal may play a role in increased LV afterload and the subsequent concentric remodeling which might ultimately lead to diastolic dysfunction, paving the way for subsequent heart failure with preserved ejection fraction. Taken together, our data suggest that increased aortic stiffness and backward flow are associated with increased EAT which could ultimately lead to identify a group of patients at higher cardiometabolic risk.

Eotaxin was correlated to backward flow parameters suggesting that eotaxin may participate to vascular inflammation. This interpretation is supported by the study by Haley and colleagues (42), which demonstrated increased eotaxin expression in atherosclerosis. We also found a significant association between CXCL5 and impaired ascending aorta flow reversal, supported by previous findings in patients with aortic aneurysm, revealing that CXCL5 was overexpressed in patients compared to controls (43). In our study, backward flow volume was also associated with other cytokines such as MIF and IP-10, which are known to be linked to inflammatory aortic pathologies, as reported in the literature (44–46).

This study has some limitations. First, the limited sample size. We matched T2D patients to healthy controls to avoid any demographic bias between the two groups. Second, this study had a cross-sectional design, and thus lacks prospective data. Finally, our findings only suggest that the relationship between increased EAT and increased aortic stiffness and flow disturbance would be explained by the fact that EAT acts like a proinflammatory adipose tissue. This observation should be confirmed in the future on a larger population while considering additional biomarkers specific to inflammation and using a longitudinal design.

## Conclusions

Aortic stiffness and flow disturbances are more pronounced in T2D patients as compared to controls and associated to increased EAT volume, which is considered as an index of cardiometabolic severity. A mechanistic relationship between EAT and aortic stiffness remains to be established in future studies.

## Data availability statement

The original contributions presented in the study are included in the article/supplementary material. Further inquiries can be directed to the corresponding author.

## Ethics statement

The studies involving human participants were reviewed and approved by the Ethics Committee CPP Ile-de France. The patients/participants provided their written informed consent to participate in this study.

## Author contributions

NK, AR, FA, JAW, EmB and KB contributed to conception and design of the study. MZ, AN, MP, TN, TD, VDV, EmB and KB analyzed the data. KB performed the statistical analysis and wrote the first draft of the manuscript. All authors contributed to article and apporved the submitted version.
